# Patients’ experience of identifying and managing exacerbations in COPD: a qualitative study

**DOI:** 10.1038/npjpcrm.2014.62

**Published:** 2014-09-18

**Authors:** Veronika Williams, Maxine Hardinge, Sara Ryan, Andrew Farmer

**Affiliations:** 1 Nuffield Department of Primary Care Health Sciences, University of Oxford, Oxford, UK; 2 Oxford Centre of Respiratory Medicine, Oxford University Hospitals NHS Trust, Churchill Hospital, Oxford, UK

## Abstract

**Background:**

Effective self-management in chronic obstructive pulmonary disease (COPD) is crucial to reduce hospital admissions and improve outcomes for patients. This includes early detection and treatment of exacerbations by patients themselves.

**Aims:**

To explore patients’ current understanding and experience of managing and identifying COPD exacerbations at home.

**Methods:**

A qualitative, interview-based study was carried out in patients’ homes. Interviews were audio-recorded, transcribed and analysed using a grounded theory approach. Forty-four patients (17 women, 27 men; age range 55–85 years), with moderate-to-very-severe COPD, were recruited to the interview study from primary and secondary care settings in Oxford, UK, during 2012–2013.

**Results:**

Patients identified exacerbations on the basis of measurable, ‘visible’ symptoms, such as cough and sputum and ‘invisible’ symptoms, such as chest sensations and bodily knowledge. Most patients seemed to use a combination of these approaches when identifying exacerbations, according to the symptoms that had the most impact on their well-being. Patients used additional self-management strategies during an exacerbation, such as self-medication (antibiotics and steroids) and monitored their recovery. Contact with health-care professionals usually occurred when patients felt no longer able to manage themselves.

**Conclusions:**

Patients use both assessment of objective biomarkers, which are aligned with medical knowledge, and subjective symptoms based on their experience, to identify and manage exacerbations of COPD. Health-care professionals and clinicians should acknowledge this ‘expert patient’ knowledge and integrate this into patients’ care plans to facilitate early recognition and treatment of exacerbations.

## Introduction

In the UK, one in three patients with chronic obstructive pulmonary disease (COPD) are readmitted within 28 days of a hospital admission for an exacerbation.^1^ Effective self-management has potential to reduce hospital admissions and improve outcomes in COPD.^2,3^ As patients are typically first to notice a change in their condition, it is crucial that they understand what constitutes an exacerbation, recognise deteriorating symptoms and react appropriately.^4^

There is still no consensus on how to define exacerbations,^5,6^ and how they are recognised and understood by patients.^7,8^ Although a previous study suggests that patients struggle with medical terminology and have poor understanding of what ‘exacerbation’ means, findings show that patients are aware of imminent exacerbations.^8^ However, the study has been criticised for its poor methodological quality.^9^ Exacerbations have previously been identified as a subjective experience and this could contribute to the ambiguity surrounding its definition.^7^ A recent meta-synthesis has provided an improved understanding of COPD exacerbations by examining the qualitative literature,^9^ yet the studies included focused on how patients experience exacerbations rather than how patients identify these.

Patients may also be reluctant to consult health-care professionals when experiencing an exacerbation^7^ and often only seek medical help when they reach a crisis point.^9^ Yet patients have reported feeling safe in treating an exacerbation at home^10^ suggesting that self-management is a viable option. Patients’ ability to identify exacerbations at an early stage is a key requirement for treating these successfully at home. Although considerable research has been published on the clinical assessment of exacerbations,^11–14^ we still have little knowledge of how patients assess and manage these.^15^ We therefore set out to identify how patients recognise and manage exacerbations at home employing qualitative methods.

## Materials and Methods

This study took place over 12 months during 2012–2013. Ethical approval was obtained from South Central Berkshire Research Ethics Committee (ref 12/SC/0437) and Research Governance was granted by Oxford Health NHS Foundation Trust and Oxford University Hospitals NHS Trust.

Patients were recruited as part of a clinical trial and a qualitative, interview-based study was conducted to explore patients’ understanding and experience of managing their COPD, including exacerbations, at home. All participants gave written consent when initially entering the trial and this was confirmed immediately before the interview.

### Patient recruitment

Patients meeting the eligibility criteria for the clinical trial (Table 1) were identified and recruited from hospital admission records, pulmonary rehabilitation programmes and general practice by a respiratory research nurse. Participants in the interview study were recruited from the trial sample using purposeful sampling strategies, whereby initially patients with different background, (such as age, gender, COPD severity, length of diagnosis, frequency and number of exacerbations), were invited to represent the variety of characteristics found within the sampled population.^16^ Sampling continued until data saturation was achieved.

### Data collection

Data were collected through in-depth interviews, which were carried out at patients’ homes. All interviews were conducted by the first author (VW) who has received extensive training in qualitative research methods. A semi-structured topic guide (see Supplementary Appendix) was used and questions focused on how patients experienced, identified and managed an exacerbation at home. Interviews lasted between 20 and 55 min. Field notes were recorded immediately after the interview to provide context of the interview and aid the analysis process. Six participants had their spouse or family member present during the interview.

### Data analysis

Interviews were audio-recorded, transcribed verbatim and anonymised transcripts were imported into NVIVO 10 (qualitative software data programme) to facilitate storage, organisation and analysis of data. Analysis followed a grounded theory approach,^17,18^ and included constant comparative method, open, axial and selective coding and memo-writing to identify theoretical links and concepts from the data.

To ensure rigour of the coding process, two interviews were coded by an experienced qualitative researcher (SR) within the department but external to the project. In addition, the researcher and the other members of the research team (VW, MH and AF) met regularly to review and discuss the analysis process and interpretation of data. Both of these processes aided transparency and credibility of findings.

## Results

Forty-four patients were recruited to the interview study and all of these agreed to take part in the interview. The characteristics of patients are given in Table 2.

The focus of this paper relates to how patients identified an exacerbation and how they managed this in addition to self-management strategies of their stable COPD.

Although patients did not use the term ‘exacerbation’, they all felt they were able to identify ‘flare ups’, and described different ways of doing so. These can be collated into two distinct approaches as shown in Table 3.

### ‘Visible’ symptoms

Patients reported a number of visible or measurable symptoms relating to an exacerbation of COPD, such as sputum, cough and temperature. For some patients, symptoms such as coughing were present even in stable periods of COPD, and were more severe during exacerbations, whereas other symptoms such as sputum production were only present during exacerbations, thus clearly marking a worsening of patients’ condition. Using these ‘visible’ symptoms to identify an exacerbation appeared very much in line with clinical assessment of biomarkers indicating an exacerbation as used by health-care professionals.

Patients also experienced limitations in their physical abilities as a result of their COPD symptoms on an everyday basis. During an exacerbation, these limitations were further worsened and rendered some patients house or even bed bound:

‘I was walking back from the kitchen and I got to the first door and then I just couldn’t get any further and I started to … my legs went and … I actually had to crawl back here’ (HA010)

Although it was predominately the increased breathlessness associated with the exacerbation of their condition that generated these severe physical limitations, some patients focused on the physical manifestation and consequences more than the experience of increased breathlessness when describing an exacerbation.

### ‘Invisible symptoms’

In addition to these visible and measurable symptoms and signs, patients also described less specific, invisible and subjective symptoms indicating an exacerbation. These related to chest sensations, such as heaviness, tightness and soreness of the chest and were perceived as the body ‘telling’ the patient that an exacerbation is developing.

The most common way patients identified an exacerbation related to bodily knowledge, whereby they ‘just knew’ that they were experiencing an exacerbation.

‘I know when I’m really bad’ (HA007)

This ‘knowing’ appeared to relate to experiential knowledge of their condition and body, where patients had learned through successive exacerbations how their body responded to an acute worsening of their COPD:

‘I’ve had so many flare-ups in the last twelve months that I know when it’s an infection’ (HA009)

This experiential knowledge of their body, symptoms and current condition also translated to patients’ self-management of an acute exacerbation.

### Exacerbation management and the experience of ‘good days and bad days’

Patients used a variety of self-management strategies during stable periods of their condition, including pacing, resting and breathing techniques. However, during an exacerbation, patients drew on additional strategies including self-medication (antibiotics and steroids), which was referred to as ‘back up medication’ as well as ‘over the counter medication’ and monitoring of recovery (Table 4). Patients talked about ‘bad’ days and ‘good’ days as part of their everyday illness experience:

‘you know, it comes and goes, you know, you can have your good days and you can have your bad days’ (PR1019)

An exacerbation signified an acute phase of worsening and escalation of their ‘bad days’.

‘I just feel rough… when I’m feeling really rough’ (GP1052).

Patients experienced COPD as a worsening condition that is often interrupted by exacerbations experienced as acute crises, where patients felt ‘really rough’ rather than the daily variability of symptoms seen as ‘good’ days and ‘bad’ days. Patients appeared to be confident in distinguishing ‘bad’ days from acute worsening indicating an exacerbation.

### Self-management strategies

Patients were keen to manage their exacerbations at home by themselves and this was their initial approach to dealing with an acute worsening of their condition. They learned from experience and took anticipatory measures to reduce the impact of COPD.

‘I’m starting to sort of look ahead and say instead of getting in the shower and then realising half way through a shower I can’t breathe …if I have a flare up and have to get out of the shower half way through my shower, covered in suds, to get my inhaler because my chest is so tight, I actually take the inhaler now before I go into the shower.’ (PR1042)

These approaches often focused on usual strategies such as ‘planning ahead’ and ‘pacing’, but these became particularly central to managing the impact of exacerbations.

### Self-medication

Once patients identified an acute exacerbation of their COPD due to a virus or bacterial infection, they would commence treatment with ‘back up medication’, such as oral steroids and antibiotics. This decision to self-medicate was often based on changes of both ‘visible’ and ‘invisible’ symptoms.

‘Usually I go by the sputum. If it changes colour, I go on antibiotics and steroids.’ (HA007)

‘When I know that it’s unbearable to breathe and if I get tight.’ (HA008)

‘I know exactly when it’s time to take them *[antibiotics]*’’ (OC1065)

Patients appeared to feel confident in identifying exacerbations and commence treatment with antibiotics and/or steroids. However, not all patients were keen on taking medication, even if they felt they had accurately identified an exacerbation. For some, there was a perception that it would be better for the body to overcome these by itself.

‘I don’t really want to take too many things if you can get better without it.’ (PR034)

Side effects of medication were also perceived as a barrier to medication adherence.

‘I think last, was it last year or the year before, I had some [*antibiotics*], well I stopped taking them because I didn’t feel too good after taking them. I felt worse’ (HA012)

‘ Because I’ve been taking so many steroids I’ve got absolutely no muscle definition at all… I can barely carry half a bucket of water for more than about 10 feet. …. But I’ve been trying not to use the steroids too much because of the, the effects that has on me. Like muscle definition’ (RN1001).

### Monitoring of recovery and contact with health-care services

Patients monitored their recovery to decide whether their symptoms responded to the treatment. When patients successfully managed their exacerbation themselves they would continue to manage their COPD through a period of recovery using their usual self-management strategies. However, patients were also aware when their condition was not responding to treatment in the usual way:

‘last Thursday I finished the course, and I was reducing the steroids and I thought I’m still not right.’ (HA007)

If this recovery process was not progressing as expected, patients initiated contact with health-care services, yet this was not necessarily a linear process of contact. Some patients initially contacted their general practitioner or respiratory nurse and others called an ambulance directly. This decision was made on the basis of the severity of symptoms and a sudden change in symptoms.

‘I know when I’m really bad and I just say to the wife, ‘phone for an ambulance’’. (HA007)

Contact with health-care professionals was often described as part of a continual communication process between patient and health-care professional:

‘The last one … I had a chest infection. Like just as I got one normally. I took my back up medication and Amoxicillin and prednisolone. Took that with a 10-day course, it seemed to be easing off. The doctor said to give me more just to wean off and in the weaning down process it came back, I phoned her and she said, ‘Oh right, we’ll change the antibiotic and put you on co-amoxiclav’ (HA015)

Patients seemed to assess their recovery period and base their decision on when to contact health-care services according to how their symptoms responded to treatment. Although some patients initially contacted their general practitioners or respiratory nurse, others often found themselves at crisis point where they would be admitted to an emergency department.

## Discussion

### Main findings

Our study demonstrates that patients identify exacerbations by assessing both visible symptoms based on clinical parameters, and invisible symptoms based on experiential knowledge. Patients who had experienced exacerbations in the past appeared to ‘just know’ when they were exacerbating and in addition to everyday self-management techniques, such as pacing and breathing techniques, self-administered antibiotics and steroids to manage this. They would monitor the course of their exacerbation and access health-care professionals when they felt there was no improvement in their condition. However, this was sometimes delayed until patients reached crisis point and had to be admitted to an emergency department.

### Interpretation of findings in relation to previously published work

Previous research found that patients often had difficulty in understanding the term ‘exacerbation’,^8^ and there is still no consensus on symptom changes that define an exacerbation.^19^ In line with existing research,^7^ we found that patients use clinical markers as a way to identify exacerbation and potentially contact health-care professionals. However, our results add to these findings by identifying that patients may not only assess exacerbations according to such specific ‘visible’ symptom changes but also use experiential knowledge to assess both objective signs and subjective ‘invisible’ chest sensations, including breathlessness and bodily changes. The invisibility of breathlessness in COPD has been reported previously and patients’ need to make this symptom visible to legitimise seeking help.^9^ The importance of acknowledging experiential knowledge in helping patients manage their illness has been identified in other long-term conditions such as diabetes.^20^ Patients with a long-term condition such as COPD utilise both clinical knowledge of objective, visible changes of their symptoms, and monitoring invisible symptoms, usually related to experiential knowledge, to identify and manage exacerbations.

During an acute phase of their COPD, patients particularly notice physical limitations and this is often found to be a more important change to patients than respiratory symptoms alone.^9^ Research into the reasons why patients contact health-care professionals during or shortly after exacerbations in COPD, found that for some, but not all patients, objective visible symptoms such as change in sputum colour is a frequent cause for health-care consultations.^7,8^ However, the emotional response, mainly fear, to the change of overall well-being was a much stronger trigger to contact NHS professionals.^9^ This emotional response could be interpreted as an intrinsic knowing that ‘something is wrong’ and patients no longer felt they were able to manage such acute crises themselves. Similarly, patients in our study monitored their condition for improvement when treating their exacerbation and if this was not successful and patients felt ‘really bad’, they would contact health-care services. This experiential knowledge appears to be an important way for patients in helping them identify, manage and monitor their exacerbations.

Patients with COPD often experience stable periods of their condition and it has been argued that they see their condition as a ‘way of life’^21^ rather than an interruption in itself, as symptoms are often present for a considerable time before diagnosis. The idea of ‘good’ days and ‘bad’ days is reflected in established frameworks of illness experience,^22^ as patients strive to minimise the impact of their condition. However, good days and bad days are an accepted variability of COPD,^9^ whereas an exacerbation presents a real interruption to how patients manage their condition themselves on a daily basis, requiring patients to self-manage on various levels, including self-medication, monitoring of symptoms and non-pharmacological management of symptoms. Although previous research has found that patients may have difficulty in differentiating between a ‘bad day’ and an exacerbation of COPD,^7,23^ participants in our study felt they were able to make this distinction. The majority of participants in our study had been living with COPD symptoms for a number of years and thus this experience may have helped them in distinguishing ‘bad days’ from an onset of an exacerbation.

### Strengths and limitations of this study

This paper reports on findings from a qualitative study with 44 patients with COPD. Although patients were recruited using purposeful and theoretical sampling strategies, the sample may not fully represent the diversity of patients with COPD as patients were recruited as part of a trial and thus had to fit certain eligibility criteria. However, within these criteria we ensured a maximum variation sample. As the clinical trial aimed to assess the efficacy of a self-management support intervention,^24^ patients recruited to the study may have been more positive and engaged in self-management than those declining to take part in this trial and thus not taking part in the embedded qualitative study. Nevertheless, our findings provide important insights into how patients with COPD identify and manage exacerbations with COPD. Qualitative research is often criticised for its subjective interpretation of data. We ensured rigour by discussing analytical processes in depth throughout the study among the research team members. In addition, 10% of transcripts were double coded by one of the co-authors (SR), who was not part of the study team, and this did not produce any conflicting findings.

### Implications for future research, policy and practice

Our findings show that patients feel they are able to identify and manage exacerbations of their COPD at home. They use both objective, clinical measures as well as subjective, invisible symptoms, based on experiential knowledge, when identifying exacerbations. As there is still no consensus on a definition of an exacerbation in COPD and health-care professionals assess patients using objective clinical features of the condition, it is important to note that patients may rely more on their experiential knowledge of subjective ‘invisible’ symptoms when identifying exacerbations. This also indicates that COPD can have an impact on patients in different ways, thus highlighting the heterogeneity of this patient group and the need for health-care professionals to consider these more diverse and less obvious symptoms that may indicate an exacerbation. This is also of particular interest when implementing interventions such as tele-monitoring, which requires patients to complete daily symptoms diaries and monitor pulse oximetry to predict and capture exacerbations in COPD, as we may not be asking patients the right questions in relation to the symptoms experienced during exacerbations.

Similarly, patients’ decision-making process in managing exacerbations was framed by both clinical assessment and experiential knowledge, as well as weighing up side-effects of medication. Patients appear confident in managing exacerbations at home and through monitoring of recovery would contact health-care services if their condition did not improve. However, such monitoring was also often based on experiential knowledge as well as clinical parameters and may differ between those patients who had substantial experience and knowledge gained through living with this condition for a number of years and attending pulmonary rehabilitation courses and those who are newly diagnosed.

Our findings suggest that patients’ experiential knowledge has an important role in how patients identify and self-manage exacerbations, and this ‘expert patient’ experience could inform current clinical practice by augmenting existing clinical assessment and measures. This may be of particular value in educating newly diagnosed patients in identifying exacerbations, where patients’ own experience of more diverse symptoms may have as much of a role as clinical features of an acute worsening of COPD. The findings from this qualitative study could therefore inform further research into quantifying these experiences to clarify current definitions of exacerbations in COPD and inform clinical measures in an attempt to allow earlier recognition and effective management of exacerbation, particularly in light of increasing implementation of tele-monitoring interventions.

### Conclusions

Patients use both assessment of objective biomarkers, which are aligned with medical knowledge, and subjective symptoms based on their experience, to identify and manage exacerbations of COPD. Health-care professionals and clinicians should acknowledge this ‘expert patient’ knowledge and integrate this into patients’ care plans to facilitate early recognition and treatment of exacerbations.

## Figures and Tables

**Table 1:** Inclusion/ exclusion criteria for interview study

A diagnosis of chronic obstructive pulmonary disease (COPD)
Aged >40 years
A forced expiratory volume in 1 s (FEV_1_) post-bronchodilator <80% and predicted ratio of FEV_1_ to forced vital capacity (FVC) <0.70
Smoking history >10 pack years
MRC dyspnoea scale ⩾2
Registered with a general practice and with an exacerbation of COPD requiring home treatment or hospital admission in the previous year, or referred for pulmonary rehabilitation
Absence of other significant lung disease
Absence of chronic heart failure defined by the New York Heart Association classification system as severe (Grade IV)
Able to give informed consent
Life expectancy of >3 months
Able to adequately understand written and verbal English

**Table 2:** Patient characteristics

	*Number of patients*
Sex (male/female)	27/17
Age (years, mean; range)	71; 55–85
	
*Severity of COPD (GOLD)*	
GOLD stage II	14
GOLD stage III	21
GOLD stage IV	9
Duration of symptoms (years; range)	1–25
Home oxygen use	11
Previous attendance of pulmonary rehabilitation programme	33
	
*Living setup*	
Living with spouse/family	29
Living alone	15

Abbreviation: COPD, chronic obstructive pulmonary disease.

**Table 3:** Patient identification of exacerbation: visible and invisible symptoms

*Measurable ‘visible’ symptoms*	
Physical symptoms	*Cough* ‘I started coughing and I know by morning I’d been coughing like every three quarters of an hour and … it’s bad’ (HA002) *Sputum production*: colour, viscosity, taste and amount. ‘Well I do, I start coughing up a bit of phlegm and that and it tastes vile and then I know I’m getting an infection.’ (HA009) ‘I knew that the … sputum was green and getting thicker’ (HA003) *Cold symptoms* ‘normally the first time I’m getting something going to the chest is I normally start to get a sore throat’ (HA015)
Physical limitations	*Functional limitations* ‘I can’t even walk from the bathroom to the bedroom without having to stop’ (HA003)
	
*Subjective ‘invisible’ symptoms*	
Chest sensations	*Soreness of chest* ‘I would say it’s my chest telling me, the soreness in my chest developing into, not pain as such but it starts feeling, see just across there, it starts feeling a bit sore there [points at chest with hand].’ (HA009) *Tight chest* ‘Get really tight down here [points at chest with hand] I mean say in all my life you have cold, the first thing it do is they get it to your chest and it gets tight.’ (HA008) *Heavy chest* ‘I start to feel, feel unwell and I can feel the weight on my chest. You can feel the stuff building up’ (HA015) *Breathlessness* ‘I couldn’t breathe, my chest went and I just couldn’t breathe’ (HA009)
Body knows	*Lack of energy* ‘generally feeling sort of rough… Lethargic. Yes. Lose the energy factor’ (HA012) *Knowing* ‘you know in your own body what’s right or wrong’ (PR032)

**Table 4:** Patients’ self-management approaches during an exacerbation of COPD

*Self-medication and self-management strategies*	*Self-monitoring*	*Contact with health-care services*
‘I try and do it myself if I can, manage it myself’ (HA007) ‘Well, there are very, it depends where you’re more comfortable, hanging onto the back of that armchair, is a favourite place… because it, because it stretches your lungs out. Or sitting here. Or [um] I have a lot of faith in the [uh] breathing exercises’ (RN1002) ‘well it’s gone on for quite a long time and so we’ve got fairly experienced at the way to treat it, you know’ (OC1045)	‘It's just there and so straight away I started taking the prednisolone and then … I think not the next day but the day after that I started taking the antibiotics, because they told me just have a couple of days on the prednisolone first to see if you can breathe easier but I knew that the … sputum was green and getting thicker’ (HA003) ‘Well if this chest don’t get any better, if it’s still the same tomorrow morning, I’ll start on the steroids and then phone him [GP] and get him to phone me back and say, ‘Look you should start on some…’ (HA009)	‘I take a week’s of what you’ve got. The antibiotic. But if it's getting close to the end of the week and I feel it’s not improved or its still there, you know, it might have improved a bit, but not enough,’ I said, ‘I always go then and make sure I get an appointment, so that if I’ve got to take more I can follow them on without having a break in between.’ (PR032) ‘take more steroids and me antibiotics that I’ve got at home, and obviously if it doesn’t get better then I go to the doctor again’ (HA001)
