# Supplementary material for: Patients’ experience of identifying and managing exacerbations in COPD: a qualitative study
Source: NPJ Prim Care Respir Med. 2014 Sep 18;24:14062–. doi: 10.1038/npjpcrm.2014.62 (PMC4498166; doi:10.1038/npjpcrm.2014.62)
Supplement: Supplementary Appendix [file npjpcrm201462-s1.pdf]

SUPPLEMENTARY APPENDIX

Interview schedule pre-trial

Introductions/ written consent/ audio recorder on

As you know we are currently developing a mobile health system to support people living with COPD to manage their own condition. We would like to find out a bit more about how you currently experience your condition, and who manages COPD at home at the moment.

1. First of all, could you tell me about how your condition affects you on a daily basis?
2. How do you currently manage your disease on a good day (self, carer, health-care professional input)?
3. Tell me a bit more about what a good day is like.
4. Do you know what an exacerbation is?
5. How do your symptoms change when you have feel an exacerbation coming on? How does it start?
6. Tell me a bit more about what a bad day looks like.
7. How do you manage your condition when you have a bad day?
8. What are the main issues that you face in managing your condition?
9. How confident do you feel in managing your COPD at home?
10. What is your experience of the current care provided to you?
11. Is there anything you can think of that might facilitate the management of your condition?
12. How do you feel about using IT in your daily life (mobile phones, computer, internet, smart phones)?
13. How do you feel about using technology to help manage your condition?
14. Can you think of any difficulties/challenges such a system might bring?
15. What do you expect from this application in terms of managing your condition?

Many thanks for taking part in this research.
